# Repeated Cycles of Binge-Like Ethanol Exposure Induces Neurobehavioral Changes During Short- and Long-Term Withdrawal in Adolescent Female Rats

**DOI:** 10.1155/2022/7207755

**Published:** 2022-10-25

**Authors:** Letícia Yoshitome Queiroz, Igor Gonçalves de Oliveira, Sabrina de Carvalho Cartágenes, Luanna Melo Pereira Fernandes, Sávio Monteiro dos Santos, Wallax Augusto Silva Ferreira, Fernando Augusto Rodrigues Mello Junior, Leonardo Oliveira Bittencourt, Edson Bruno Campos Paiva, Rommel Mario Rodríguez Burbano, Edivaldo Herculano Correa de Oliveira, Marta Chagas Monteiro, Rafael Rodrigues Lima, Enéas Andrade Fontes-Júnior, Cristiane do Socorro Ferraz Maia

**Affiliations:** ^1^Laboratory of Pharmacology of Inflammation and Behavior, Faculty of Pharmacy, Institute of Health Science, Federal University of Pará, Belém, Pará, Brazil; ^2^Program of Post-Graduation of Pharmaceutical Science, Institute of Health Science, Federal University of Pará, Brazil; ^3^Physiological and Morphological Sciences Department, Biological and Health Science Centre, State University of Pará, Belém, Pará, Brazil; ^4^Laboratory of In Vitro Assays, Immunology and Microbiology, Faculty of Pharmacy, Institute of Biological Sciences, Federal University of Pará, Belém, Pará, Brazil; ^5^Laboratory of Cytogenomics and Environmental Mutagenesis, Environment Section (SAMAM), Evandro Chagas Institute (IEC), Ananindeua, Pará, Brazil; ^6^Faculty of Natural Sciences, Institute of Exact and Natural Sciences, Federal University of Pará (UFPA), Brazil; ^7^Laboratory of Molecular Biology, Ophir Loyola Hospital, Belém, Pará, Brazil; ^8^Laboratory of Functional and Structural Biology, Biological Science Institute, Federal University of Pará, Belém, Pará, Brazil; ^9^Federal University of Western Pará-UFOPA, Santarém, Pará, Brazil

## Abstract

Alcohol consumption is spread worldwide and can lead to an abuse profile associated with severe health problems. Adolescents are more susceptible to addiction and usually consume ethanol in a binge drinking pattern. This form of consumption can lead to cognitive and emotional disorders, however scarce studies have focused on long-term hazardous effects following withdrawal periods after binge drinking in adolescents. Thus, the present study aims at investigating whether behavioral and cognitive changes persist until mid and late adulthood. Female Wistar rats (9-10 animals/group) received intragastric administration of four cycles of ethanol binge-like pattern (3.0 g/kg/day, 20% w/v; 3 days-on/4 days-off) from 35^th^ to 58^th^ days old, followed withdrawal checkpoints 1 day, 30 days, and 60 days. At each checkpoint period, behavioral tests of open field, object recognition test, elevated plus maze, and forced swimming test were performed, and blood and hippocampus were collected for oxidative biochemistry and brain-derived neurotrophic factor (BDNF) levels analysis, respectively. The results demonstrated that adolescent rats exposed to binge drinking displayed anxiogenic- and depressive-like phenotype in early and midadulthood, however, anxiety-like profile persisted until late adulthood. Similarly, short-term memory was impaired in all withdrawal periods analysed, including late adult life. These behavioral data were associated with oxidative damage in midadulthood but not BDNF alterations. Taken together, the present work highlights the long-lasting emotional and cognitive alterations induced by ethanol binge drinking during adolescence, even after a long period of abstinence, which might impact adult life.

## 1. Introduction

Alcohol has been used in different cultures, religious, and social practices [1]. Alcohol consumption can lead to abuse profile, which is associated with episodes of anxiety, depression, insomnia, and suicide, in addition to severe health problems, such as heart disease and liver cirrhosis [2]. Adolescent individuals are more susceptible to alcohol abuse, since this period of life is characterized by increased risk-taking behavior, high exploration levels, novelty, and sensation-seeking phenotype [3–6]. Moreover, adolescents consume higher levels of alcohol per occasion [7], which characterizes the binge drinking pattern [8]. Binge drinking paradigm has been described as an alcoholic consume that reaches blood alcohol concentration level about 0.08 g/dL, equivalent to four or more drinks for women, and five or more drinks for men for two hours [8].

Surprisingly, adolescents ranging from 15 to 19 years old consists of current drinkers, which the prevalence of alcohol use among 15-year-old students reaching values of 50%-70%, with consumption by females increasing worldwide [1, 9, 10]. Female adolescent individuals represent a relevant risk group, due to greater vulnerability to adverse effects and risk of behavioral deficits related to their counterparts [11, 12]. Actually, studies have demonstrated that females have more susceptibility to acute and long-term alterations of mood and memory as well as higher vulnerability to neuroinflammation induced by ethanol exposure [13–15].

Adolescent limbic regions, which includes hippocampus, amygdala, nucleus accumbens, prefrontal, frontal and orbital frontal cortices, and hypothalamus, present particular vulnerability to alcohol effects, reflecting profound brain maturational changes period, such as dendritic arborization, synaptic pruning, and myelination processes [3, 16]. Thus, acute and chronic ethanol consumption induce structural, physiological, and functional changes in central nervous system (CNS). These modifications can display neurodegenerative processes promoting neuronal death and, consequently, neurobehavioral changes, such as learning and memory deficits, anxiety phenotype, and motor impairments [17, 18]. In intermittent binge-like ethanol consumption, immediate hazardous outcomes (i.e., memory impairments), and long-term consequences (i.e., anxiety and sleep disruption) have been recorded [19, 20].

In agreement with this, our group has focused on ethanol exposure challenge in adolescent female models. We found that heavy chronic ethanol intoxication (6.5 g/kg/day for 55 days) during adolescence induced motor incoordination, spontaneous locomotor disruption, muscle strength impairment, bradykinesia on motor domain, related to neuronal loss, astrocytic activation, morphological changes, and oxidative damage on motor-related brain regions [18, 21–23]. In addition to motor disruption, heavy chronic ethanol paradigm during adolescence also elicited emotional and cognitive behavioral alterations, as anxiety-like features and short-term memory impairment, associated to histological hippocampal alterations [24]. In binge-drinking adolescence challenge, we found that motor, cognitive, and emotional domains were negatively affected, including with long-term repercussions in anxiogenic phenotype, even after 14 days of withdrawal [25–30]. Thus, considering these intriguing data above and the gap in literature about the long-lasting consequences related to binge-like adolescence ethanol consume, we ask whether such behavioral and cognitive alterations might persist until midadulthood. We now characterized the short- and long-lasting detrimental effects elicited by ethanol binge-drinking paradigm during adolescence on emotional and cognitive domains in female rat.

## 2. Materials and Methods

### 2.1. Animals

Adolescents female *Wistar* rats (*n* = 60; 21 days old) were obtained from Animal Facility of the Federal University of Pará and maintained on a 12 : 12 h light/dark cycle (lights on 7 : 00 AM; five animals/cage), with food and water *ad libitum,* until the beginning of binge-like protocol administration (35 days old). All procedures were approved by Ethics Committee on Experimental Animals of the UFPA (license number 1821040417) and followed NIH guidelines for the Care and Use of Laboratory Animals. Female rats were chosen based on previous studies of our laboratory that established that binge drinking exposure during adolescence induces emotional and cognitive impairments in female rats [24, 26], as well as previous study which reported that ethanol-induced brain injury is more evident in female than in male subjects [15].

### 2.2. Ethanol Binge-Drinking Protocol and Experimental Groups

**Figure 1:**
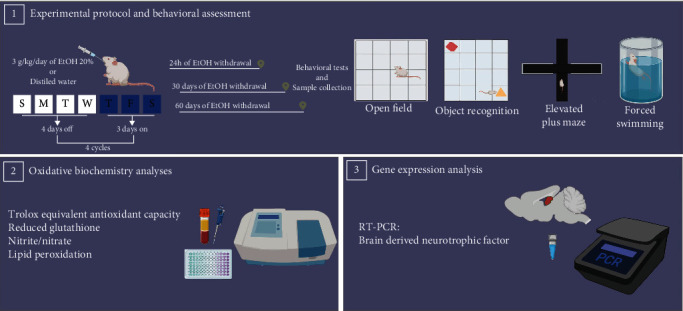
Experimental design.

Intragastric administration of ethanol (3.0 g/kg/day, 20% w/v ethanol) in four cycles of binge-like pattern (3 days-on/4 days-off) or distilled water was employed from 35^th^ to 58^th^ days old [26] (Figure 1). According to our previous and validated results, this ethanol protocol exposure reaches a blood alcohol concentration of 237.6 ± 16.76 mg/dL and 297.3 ± 34.49 mg/dL following one cycle and four cycles of binge drinking exposure, respectively [26]. These values reach >80 mg/dL, confirming the binge-drinking model employed (NIAAA, 2004).

Following binge-drinking cycles administration, both ethanol and control groups were subdivided in 3 periods of withdrawal assessment, i.e., 1 (59^th^ days old), 30 (88^th^ days old), and 60 (118^th^ days old) days [31]. The withdrawal periods were chosen to assess the early, mid, and late adulthood perspective [32–35].

### 2.3. Behavioral Testing

As mentioned above, behavioral tests were performed at 1, 30, and 60 days following the 4 binge-like ethanol cycle. Firstly, animals were habituated in a sound-attenuated room under low-intensity light (12 lux) for 1 h. Then, open field, object recognition, elevated plus maze, and forced swimming tests were conducted between 08 : 00 AM to 4 : 00 PM in a behavior sequential battery with an interval of 3 minutes between each task (Figure 1). Behavioral assays were videotaped for further analysis.

#### 2.3.1. Open Field

Open field apparatus was used to evaluate anxiety-like behavior under spontaneous exploratory activity [33, 36]. Briefly, rats were placed in the center of an acrylic black squared arena (100 × 100 × 40 cm), and free exploitation was permitted for 5 minutes. Central distance traveled and rearing were recorded as indicatory of anxiety-like behavior [37, 38].

#### 2.3.2. Object Recognition Test

Thirty minutes after open field test, object recognition test was carried out in arena, since in a familiar environment rodents present preference for new objects [39]. Thus, animals were submitted to training section, which animals were exposed to two identical objects positioned in opposite corners of the arena for 3 minutes (10 cm from the wall and 70 cm distance between them). Thirty minutes following training phase, test phase was employed, introducing the animal in arena with both devices, a new object and a familiar one exactly at previous localization for 3 minutes. Exploration time spent (i.e., less than 4 cm from the snout to the object) at each object was recorded.

Analyses were performed considering total exploration time spent on the two objects in the training phase (T1 + T2). Discrimination index was defined by the difference in the exploration time between the new object (TN) and the familiar device (TF), divided by the total time spent exploring between the same objects in the test phases, as follows: (*TN* − *TF*)/(*TN* + *TF*) [26].

#### 2.3.3. Elevated Plus Maze

Elevated plus maze is a wooden plus apparatus, elevated 50 cm from the floor, with opposite two closed arms (50 × 10 × 40 cm) and two open arms (50 × 10 × 1 cm), surrounding by 1 cm device protection to prevent animals fall validated for anxiety-like assessment [40]. Animals were individually placed in the center of the apparatus facing the enclosed arms, which freely exploratory activity for 5 minutes [41, 42]. Percentual of open arms entries (% OAE) and open arms time (%OAT) were measured according to the formula [*OA*(*E*/*T*)/*OA*(*E*/*T*) + *closed* *arms* *entries* − *CA*(*E*/*T*)] × 100] [26, 41, 43].

#### 2.3.4. Forced Swimming Test

Forced swimming test was employed following elevated plus maze assay. Animals were individually inserted in a forced swim apparatus, which consists of an acrylic cylinder (30 cm diameter × 50 cm height), filled with water (40 centimeters of volume) at a temperature of 23 ± 1° C for 5 minutes. This test is used to measure depressive-like behavior, by immobility time parameter recorded in the last three minutes of the test [24, 44–46].

### 2.4. Biochemical Analyses

After behavior assessment, animals were submitted to euthanize by cervical dislocation for biological samples collect. Blood samples were assessed by intraventricular puncture of half part of animals per group and maintained in tubes containing ethylenediaminetetraacetic acid (EDTA); posteriorly centrifugation (1400 × g for 10 min) was employed to obtain serum samples. In addition, hippocampus was dissected and mixed with RNA later in an adequate tube to evaluate brain-derived neurotrophic factor (BDNF) content. All biological samples were stored at −80°C.

#### 2.4.1. Oxidative Biochemistry in Blood

*(1) Determination of Total Antioxidant Capacity (TEAC)*. Determination of total antioxidant capacity was performed employing the antioxidant capacity equivalent to Trolox (TEAC) protocol, which consists of a colorimetric technique based on the reaction between ABTS (2,2′-Azino-bis(3-ethylbenzothiazoline-6-sulfonic acid) diammonium salt; Sigma-Aldrich, Germany) with potassium persulfate (K2S2O8; Sigma-Aldrich, Germany), directly producing ABTS ^+ •^ radical, a green/blue chromophore [47–49]. Trolox (6-hydroxy-2,5,7,8-tetramethylchromono-2-carboxylic acid; Sigma Aldrich, Germany) is a potent water-soluble antioxidant analog of vitamin E. Antioxidants presented in biological sample to this mean reduces this radical to ABTS with deviation of chemical reaction to left, on a scale dependent of the antioxidant capacity, concentration of antioxidants, and duration of the reaction, measured by spectrophotometry absorbance at 734 nm for 5 minutes. Results were expressed in *μ*M/mL.

*(2) Determination of Reduced Glutathione (GSH)*. Determination of reduced glutathione (GSH) concentrations was carried out according to Ellman method [50]. This technique is based on GSH's ability to reduce 5,5-dithiobis-2-nitrobenzoic acid (DTNB) (Sigma-Aldrich, Germany) to 5-thio-2-nitrobenzoic acid (TNB), which has been quantified by spectrophotometry at a wavelength of 412 nm [49].

Briefly, the samples were deproteinized with 2% trichloroacetic acid, which the supernatant was collected for analysis, after centrifugation at 3000 rpm for 5 min. A supernatant aliquot (20 *μ*L) was mixed with 3 mL of phosphate buffered saline (PBS)/EDTA buffer plus 20 *μ*L of distilled water for the first sample reading (T0). Then, 100 *μ*L of DTNB was added to the mixture for the second sample reading (T3) after 3 min. The difference between absorbances measured (T3 –T0) is adopted as proportional to GSH concentration, expressed in *μ*M/mL.

*(3) Determination of Nitrite/Nitrate*. Nitric oxide (NO) is highly unstable molecule, which is quickly converted to nitrates (NO3) and nitrites (NO2). Thus, the determination of its concentrations is carried out indirectly by Griess method, which measures NO2 content in sample [51]. Biological samples were deproteinized by zinc sulfate (15 g/L). In addition, centrifugation (3000 rpm) was employed for 15 minutes. Then, 100 *μ*L of sample was added to 100 *μ*L of Griess reagent (50 *μ*L of sulfanilamide solution 1% plus 50 *μ*L of N-naphthyl-ethylenediamine solution 0.1% in H3PO4 2.5%). The reaction of nitrite present in the sample elicits a pink chromogen, which is detected by a plate reader at a wavelength of 540 nm [24]. Results were expressed in *μ*M.

*(4) Determination of Malondialdehyde (MDA)*. Malondialdehyde reaction product has been correlated to lipid peroxidation [52]. Primarily based on the reaction of malondialdehyde (MDA) plus thiobarbituric acid reactive substances (TBARS; TBA; Sigma-Aldrich T5500). TBA (10 nM) plus serum sample (500 *μ*L) were submitted to water bath (pH 2.5, 94°C for 60 min) to generate MDA-TBA complex that consists of a pink-colored product. Then, samples were submitted to cooling for 10 minutes, following an additional 2 mL of butyl alcohol, vigorous homogenization in a vortex device, and centrifugation at 2500 rpm for 10 minutes. Finally, supernatant was assessed and submitted to spectrophotometric reading at absorbance of 535 nm ([53] adapted from [54]). High concentrations of TBARS have been used as an indicator of oxidative stress. Results were expressed in *μ*M/L.

#### 2.4.2. Brain-Derived Neurotrophic Factor (BDNF) Assessment by Reverse Transcription qPCR (RT-qPCR)

Firstly, total RNA was assessed. According to manufacturer instruction, tissue samples were homogenized in liquid nitrogen and submitted to chemical extraction by a RiboPure™ – Blood Kit (Ambion, USA) and treated with RNase-free DNase I. Total RNA concentration was determined by fluorimetry using the Qubit™ RNA BR Assay kit (Invitrogen, USA). The cDNA was immediately synthesized using a High-Capacity cDNA Reverse Transcription kit (Applied Biosystems, USA). GoScript™ Reverse Transcription System (Promega Corporation) was used, following the manufacturer's instructions for cDNA synthesis. Real-time PCR (qPCR) was performed using GoTaq® Probe qPCR Master Mix (Promega Corp.), as described previously [55]. All reactions were carried out in triplicate in 96-well PCR plates, using CFX96 Touch™ Real-Time PCR Detection System (Bio-Rad, USA). Data analysis was performed employing the Bio-Rad CFX Manager™ 3.1 software (Bio-Rad). MIQE guidelines was adopted, which the expression levels were normalized by *β*-actin (Actb) in control samples. Then, the relative gene expression was measured applying the formula 2 − ΔΔCT (*p* < 0.05). The expression of Bdnf and Actb was evaluated through Taqman® gene expression assays (Applied Biosystems, USA) (Rn02531967 and Rn00667869, respectively).

### 2.5. Statistical Analysis

Firstly, data normal distribution was assessed by Shapiro-Wilk test for behavioral analyzes. Results were expressed as mean ± S.E.M. of 9-10 animals/group for behavioral tests, 3-4 animals/group for biochemical oxidative assay, and 3 animals/group for tissue BDNF content. Statistical analyses were performed by Student *t*-test. *p* < 0.05 was adopted as statistically significant. BDNF levels were expressed as percentual of control. All statistical analyses were performed by GraphPad Prism 8.0 software.

## 3. Results

### 3.1. Adolescent Ethanol Binge-Like Challenge Elicits Persistent Anxiogenic-Like Behavior in Late Adulthood

**Figure 2:**
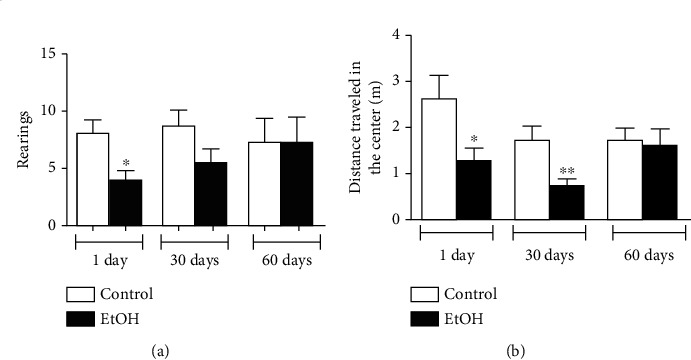
Anxiety-like behavior assessment displayed by 1, 30, and 60 days of withdrawal after binge-like ethanol treatment during adolescence on open field test. (a) Rearing number and (b) distance traveled in the center were measured. Results were expressed as mean ± SEM of *n* = 10 animals per group. ^∗^*p* < 0.05; ^∗∗^*p* < 0.01. Student *t*-test.

Anxiogenic-like features were assessed through two paradigms, open field and elevated plus maze tests. In arena, animals submitted to ethanol binge drinking paradigm exhibited reduction in rearing number following 1 day of withdrawal (*p* < 0.05; Figure 2(a)). In addition, 1 day-abstinence subjects reduced the distance traveled in the center of apparatus (*p* < 0.05; Figure 2(b)), which suggests anxiogenic-like phenotype.

Surprisingly, reduced central distance traveled was revealed by animals submitted to 30 days of ethanol binge drinking abstinence (*p* < 0.01; Figure 2(b)).

**Figure 3:**
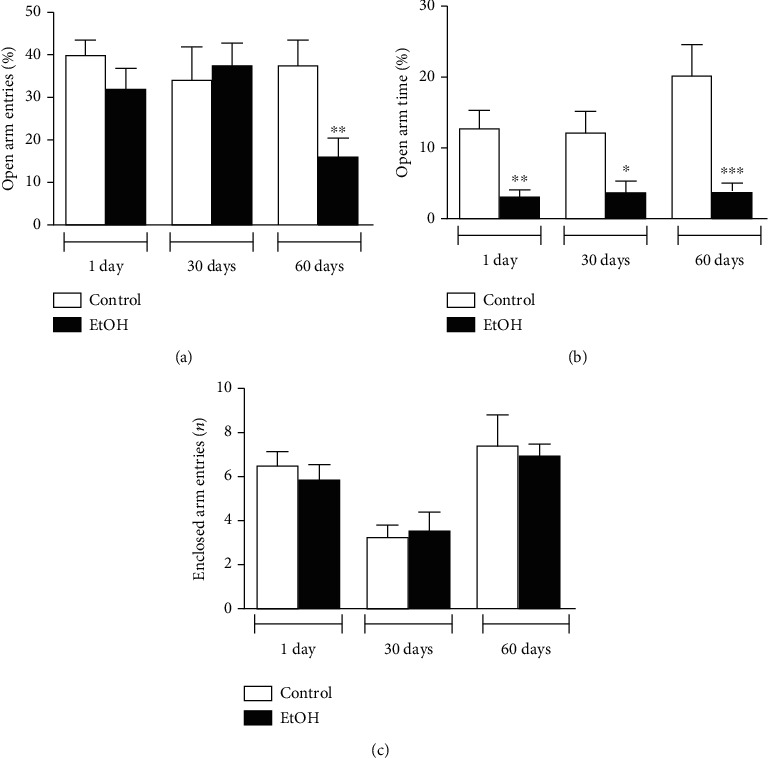
Anxiety-like behavior assessment displayed by 1, 30, and 60 days of withdrawal after binge-like ethanol treatment during adolescence on elevated plus maze test. (a) Percentage of open arms entries and (b) time and (c) number of enclosed arms entries were recorded. Results were expressed as mean ± SEM of *n* = 10 animals per group. ^∗^*p* < 0.05; ^∗∗^*p* < 0.01; ^∗∗∗^ *p* < 0.001. Student *t*-test.

In elevated plus maze test, the data obtained in arena were reinforced. The 1-day abstinent animals diminished the percentual of open arms time, but not open arms entries (Figures 3(a) and 3(b)). After 30 days of binge drinking withdrawal, reduced time spent on open arms was found (*p* < 0.05; Figure 3(b)). Of interest, 60 days of ethanol abstinence, subjects still exhibited reduced open arms entries (*p* < 0.01; Figure 3(a)) and time (*p* < 0.001; Figure 3(b)), which suggests a persistent long-lasting anxiogenic-type phenotype. Enclosed Arms entries were not modified in all checkpoints recorded (Figure 3(c)).

### 3.2. Adolescent Ethanol Binge-Like Protocol Induces Depression-Like Behavior That Persists Until 30 Days of Withdrawal but Recovers within 60 Days of Abstinence

**Figure 4:**
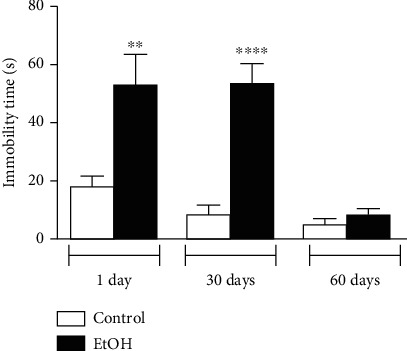
Depressive-like behavior assessment displayed by 1, 30, and 60 days of withdrawal after binge-like ethanol treatment during adolescence on forced swimming test. Immobility time (seconds) was recorded. Results were expressed as mean ± SEM of *n* = 9 − 10 animals per group. ^∗∗^*p* < 0.01; ^∗∗∗∗^ *p* < 0.0001. Student *t*-test.

In forced swimming test, ethanol-treated animals increased immobility time after 1 day (*p* < 0.01), and 30 days (*p* < 0.0001) of abstinence, related to their counterparts (Figure 4), which suggests early and long-term depressive-like behavior in early- and midadulthood. Furthermore, 60-day-withdrawal group exhibited no significant differences in immobility time, which reflects recovery of depressive-like behavior (*p* > 0.05; Figure 4).

### 3.3. Adolescent Ethanol Binge-Like Consume Induces Long-Lasting Short-Term Memory Impairment, Which Persists Until Late Adulthood

**Figure 5:**
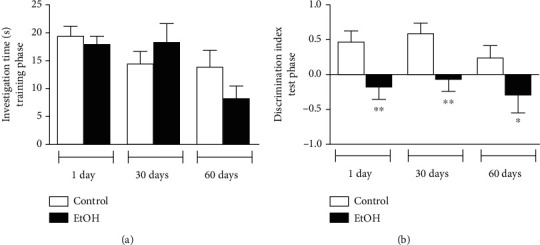
Short-term memory assessment in animals submitted to 1, 30, and 60 days of withdrawal after binge-like ethanol treatment during adolescence on object recognition test. (a) Investigation time (seconds) in training phase and (b) discrimination index in test phase were recorded. Results were expressed as mean ± SEM of *n* = 9 − 10 animals per group. ^∗^*p* < 0.05; ^∗∗^*p* < 0.01. Student *t*-test.

In object recognition test, training phase was equally performed by all tested groups (*p* > 0.05; Figure 5(a)), demonstrating that all groups effectively explored the inserted objects in training phase. However, in test stage, Student *t*-test revealed that discrimination index was reduced in all checking period (1 day: *p* < 0.01; 30 days: *p* < 0.01; 60 days: *p* < 0.05; Figure 5(b)), which confirms that short-term memory recovery was not achieved in late adulthood.

### 3.4. Adolescent Ethanol Binge-Like Treatment Negatively Interferes on Peripherical Antioxidant System and Promotes Long-Lasting Oxidative Stress in Midadulthood

**Figure 6:**
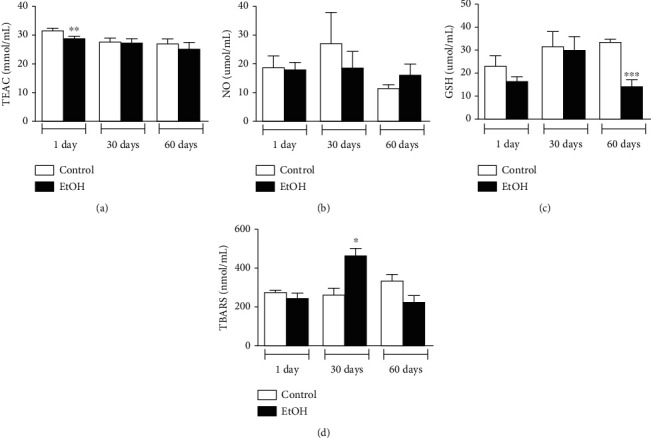
Oxidative biochemistry analysis of blood samples from animals submitted to 1, 30, and 60 days of withdrawal after binge-like ethanol treatment during adolescence. (a) Total Antioxidant Capacity (TEAC), (b) nitrite concentration (NO), (c) reduced glutathione (GSH) levels, and (d) lipid peroxidation (thiobarbituric acid reactive substances (TBARS) levels. Results were expressed as mean ± SEM of *n* = 3-4 animals per group. ^∗^*p* < 0.05; ^∗∗^*p* < 0.01; ^∗∗∗^ *p* < 0.001. Student *t*-test.

Regarding antioxidant parameters, ethanol binge-like administration decreases peripherally TEAC levels in immediate ethanol withdrawal (*p* < 0.01; Figure 6(a)), but not persist in mid nor late adulthood (*p* > 0.05; Figure 6(a)).

In GSH levels evaluation, Figure 6(b) shows that ethanol protocol administration reduced this parameter in late adulthood (*p* = 0.0005), but not in early- or midadulthood (*p* > 0.05).

Oxidative parameters demonstrated that NO levels were not modified by binge-drinking paradigm in all withdrawal periods tested (Figure 6(c)), however, lipid peroxidation was detected in 30-day-withdrawal group (*p* = 0.0054; Figure 6(d)). Such oxidative damage was recovered in 60-day-withdrawal subjects (*p* > 0.05; Figure 6(d)).

**Figure 7:**
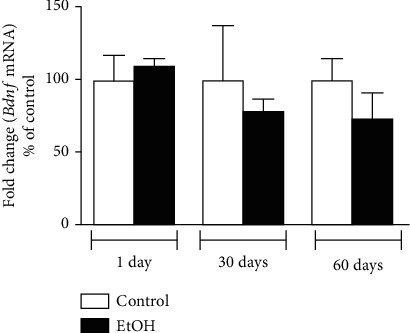
Brain-derived neurotrophic factor (BDNF) levels analysis of hippocampal tissue samples from animals submitted to 1, 30, and 60 days of withdrawal after binge-like ethanol treatment during adolescence. Results were expressed as mean ± SEM of *n* = 3 animals per group. Student *t*-test.

Finally, hippocampal BDNF evaluation was not altered in all experimental groups (Figure 7).

## 4. Discussion

Our group has focused our efforts to investigate ethanol hazardous effects during adolescence. Fundamentally through two paradigms, i.e., heavy chronic exposure and binge drinking intermittent administration, we found motor function, emotional-like disorders, and cognitive impairment related to brain areas alterations [18, 21–30]. In addition, peculiar behavioral and histological alterations (i.e., anxiety-like profile and astrogliosis) still were present after long-term abstinence (14 consecutive days) [26]. These data elicited numerous issues, which we asked whether the binge-drinking paradigm deleterious effects remain at different periods of adult life in female rats. In the present work we demonstrated for the first time that binge drinking consume during adolescence may impact on early, mid, and late adulthood, depending on the behavioral domain, associated to biochemical repercussion.

Regarding emotional behavior, we investigated anxiety- and depressive-like phenotype. Firstly, we assessed anxiety-like behavior through two different paradigms. In arena, rearing and distance traveled in the center were reduced in the 1-day- withdrawal group, which suggest anxiety-like phenotype [26, 38]. This finding was roughly confirmed by elevated plus maze assay, which open arms time was reduced in 1-day-withdrawal subjects. Depressive-like behavior was assessed by forced swim test. Our findings show that allied too anxiety-type behavior, 1-day withdrawal also exhibited depressive-like features. In fact, robust literature has postulated that immediate withdrawal has been associated to negative behavioral effects, such as dysphoria, irritability, anxiety, and depression [24, 56]. In addition, these negative emotional profiles can persist for considerable periods of time [57]. Other studies have shown similar effects in adult female rodents [26, 58] as well as adult male rats [59–62].

To evaluate whether binge-drinking immediate disorders were recovered through following withdrawal, we investigated a 30-day withdrawal, which encompasses midadulthood (88^th^ days old); as well as 60-day withdrawal, reflecting the late adulthood (118^th^ days old) [35].

In midadulthood followed adolescence ethanol binge drinking exposure, anxiety-like behavior still persists in both behavioral apparatuses. Of note, depressive-like profile also was seen at this period. These intriguing findings highlight the long-lasting emotional detrimental consequences of ethanol binge-drinking during adolescence (35-58 days old), even without additional ethanol exposure. Furthermore, anxiety-like profile was maintained at late adulthood (60-day withdrawal), but not depressive features. All these data suggest that anxiety-like behavior elicited by ethanol paradigm during adolescence can be unrecoverable, which deserves further investigation.

In agreement with our data, clinical studies have proposed that an elevate percentual of human withdrawal symptoms may persist for long-lasting periods, including emotional disorders [63, 64].

In fact, some studies showed that rats exposed to ethanol during adolescence presented high levels of dopamine in nucleus accumbens, which abstinence induces a reduction of this neurotransmitter on this area of CNS, which partially explains our data related to immediate abstinence [65–67]. We believe that long-term binge drinking exposure in critical periods of neurodevelopment may interfere with fundamental pathways linked to emotional homeostasis, which may display long-term psychiatry disorders.

In cognitive domain, adolescent binge-like administration elicited short-term memory impairment at immediate abstinence. Such data already had been extensively demonstrated by our group and others [26, 68, 69]. However, such short-term impairment phenotype was also seen in mid and late adult life. These astonished data highlight the long-lasting hazardous effects displayed by ethanol exposure in adolescence period. Vetreno and Crews [70] demonstrated that after administration in a binge drinking model from adolescence until the adult phase, after 165 days of withdrawal, the hippocampal neurogenesis is reduced in relation to control subjects, raising the hypothesis that ethanol consumption in a binge drinking pattern leads to a reduction in neurogenesis, enhanced by neuroimmune mechanisms. In addition, the authors claim that these hazardous molecular events might lead to hippocampal dysfunctions, functionally reflecting as impaired memory, even after 60 days of abstinence found in the present study. Besides, the literature shows that the hippocampus is a vulnerable region to neurotoxic effects induced by ethanol exposure, especially during adolescence [11, 71].

Emotional and cognitive domains depend on multiples molecular events. In fact, extensive literature has demonstrated that neurotrophic factors alteration and oxidative stress play a role in the pathophysiological mechanisms induced by ethanol exposure. Thus, we verified the oxidative biochemistry in plasma as well as BDNF in hippocampus. We found that TEAC levels were solely reduced one day after the last binge drinking administration immediate withdrawal). In midadulthood, lipid peroxidation was found that reflects an important mechanism of systemic disorder, including in CNS functions (for review see [72]). Oliveira et al. [26] also did not find differences between groups in the MDA levels after 4 cycles of binge drinking exposure during adolescence as well as 14 days of withdrawal, corroborating with our findings. Such MDA result was recovered in 60-day- withdrawal subjects. However, peripherical GSH levels were reduced in 118 day-old animals, which we infer that this antioxidant molecule might be acting on oxidative balance to maintain homeostasis [73], since GSH is probably the most important nonenzymatic antioxidant present in cell, a protein involved in antioxidant defense against the toxic effects of reactive species from xenobiotic metabolism, as well as an important molecule to reduce both free radicals and acetaldehyde produced by alcohol metabolism [74, 75].

MDA is an important biomarker of oxidative stress related to lipid peroxidation, which polyunsaturated fatty acids in phospholipids of cellular membranes suffers a reaction with oxygen to produce lipid hydroperoxides (LOOH) [76]. However, Fernandes et al. and Monotliu et al. found increased levels of MDA and NO in tissue and liver after ethanol treatment [27, 77], suggesting that oxidative damage in blood might not exclude histological oxidative stress. Thus, the involvement of oxidative damage could not be totally excluded in emotional and cognitive disorders described in late adulthood.

In addition, hippocampal BDNF levels were not altered in immediate, mid, and late adulthood following adolescent ethanol exposure. Conversely, Oliveira et al. [26] found reduced hippocampal BDNF immediately after ethanol bioavailability (7.5 hours), however, BNDF returned to homeostasis levels following 14 days of abstinence. We hypothesize that hippocampal BNDF levels reduce in the immediate ethanol exposure bioavailability, however, compensatory mechanisms increase BDNF normal levels in hippocampus following 24 hours of abstinence, which positively impact on mood and cognitive function. Taken together, our theory relies on that mood and cognitive disorders reported at this present work may involve other pathophysiological mechanisms but not BDNF via.

Therefore, the present study highlights that after binge-like ethanol treatment from adolescence to adulthood in female rat, mood disorders and memory impairment were found following 1 day, 30 days, and 60 days of withdrawal. In addition, anxiety- phenotype and cognitive impairment still persisted on late adulthood (60 days of abstinence). Such behavioral alterations were related to peripherical oxidative stress in midadulthood.

## 5. Conclusion

These findings revealed that binge drinking exposure during adolescence till adulthood, which consists of a vulnerable phase of brain maturation, displays long-lasting negative emotional phenotype and short-term memory disorders in mid and late adult life in female rats. These behavioral alterations were accompanied of oxidative damage in midadulthood. Of note, anxiety-like behavior and cognitive impairment were not recovery, even after long-term (60 days) withdrawal. These unprecedented evidence alerts the traditional social risk behavior involved in adolescents conduct, which can impact their adult life. Therefore, further studies are essential to assess the probable pathophysiological mechanisms implied in these long-lasting disorders.

## Data Availability

The behavioral and biochemical data used to support the findings of this study are available from the corresponding author upon request.

## References

[1] World Health Organization (2018). *Global Status Report on Alcohol and Health*.

[2] Schuckit M. A. (2019). Alcohol-use disorders. *The Lancet*.

[3] Crews F., He J., Hodge C. (2007). Adolescent cortical development: a critical period of vulnerability for addiction. *Pharmacology, Biochemistry, and Behavior*.

[4] Arain M., Haque M., Johal L. (2013). Maturation of the adolescent brain. *Neuropsychiatric Disease and Treatment*.

[5] Ehlers C. L., Criado J. R., Wills D. N., Liu W., Crews F. T. (2011). Periadolescent ethanol exposure reduces adult forebrain ChAT+IR neurons: correlation with behavioral pathology. *Neuroscience*.

[6] Fabio M. C., Nizhnikov M. E., Spear N. E., Pautassi R. M. (2014). Binge ethanol intoxication heightens subsequent ethanol intake in adolescent, but not adult, rats. *Developmental Psychobiology*.

[7] Spear L. P. (2018). Author correction: effects of adolescent alcohol consumption on the brain and behaviour. *Nature Reviews. Neuroscience*.

[8] National Institute on Alcohol Abuse and Alcoholism–NIAAA (2004). *National Institute on Alcohol Abuse and Alcoholism. Featuring Information from the National Institute on Alcohol Abuse and Alcoholism*.

[9] Laranjeira R., Madruga C. S., Pinsky I., Caetano R., Mitsuhiro S. S., Castello G. (2014). *II São Paulo: Instituto Nacional de Ciência e Tecnologia para Políticas Públicas de álcool e outras drogas Instituto (INPAD)*.

[10] Brasil. Ministério da Saúde (2018). *Vigitel Brasil 2017: vigilância de fatores de risco e proteção para doenças crônicas por inquérito telefônico*.

[11] Guerri C., Pascual M. (2010). Mechanisms involved in the neurotoxic, cognitive, and neurobehavioral effects of alcohol consumption during adolescence. *Alcohol*.

[12] Petit G., Kornreich C., Verbanck P., Campanella S. (2013). Gender differences in reactivity to alcohol cues in binge drinkers: a preliminary assessment of event-related potentials. *Psychiatry Research*.

[13] Hartley D. E., Elsabagh S., File S. E. (2004). Binge drinking and sex: effects on mood and cognitive function in healthy young volunteers. *Pharmacology, Biochemistry, and Behavior*.

[14] Pascual M., Montesinos J., Marcos M. (2017). Gender differences in the inflammatory cytokine and chemokine profiles induced by binge ethanol drinking in adolescence. *Addiction Biology*.

[15] Alfonso-Loeches S., Pascual M., Guerri C. (2013). Gender differences in alcohol-induced neurotoxicity and brain damage. *Toxicology*.

[16] Welch K. A., Carson A., Lawrie S. M. (2013). Brain structure in adolescents and young adults with alcohol problems: systematic review of imaging studies. *Alcohol and Alcoholism*.

[17] Pascual M., Blanco A. M., Cauli O., Miñarro J., Guerri C. (2007). Intermittent ethanol exposure induces inflammatory brain damage and causes long-term behavioural alterations in adolescent rats. *The European Journal of Neuroscience*.

[18] Oliveira G. B., de Andrade Fontes E., de Carvalho S. (2014). Minocycline mitigates motor impairments and cortical neuronal loss induced by focal ischemia in rats chronically exposed to ethanol during adolescence. *Brain Research*.

[19] Crews F. T., Vetreno R. P., Broadwater M. A., Robinson D. L. (2016). Adolescent alcohol exposure persistently impacts adult neurobiology and behavior. *Pharmacological Reviews*.

[20] Kyzar E. J., Floreani C., Teppen T. L., Pandey S. C. (2016). Adolescent alcohol exposure: burden of epigenetic reprogramming, synaptic remodeling, and adult psychopathology. *Frontiers in Neuroscience*.

[21] Teixeira F. B., Santana L. N. S., Bezerra F. R. (2014). Chronic ethanol exposure during adolescence in rats induces motor impairments and cerebral cortex damage associated with oxidative stress. *PLoS One*.

[22] Fontes-Júnior E. A., Maia C. S., Fernandes L. M. (2016). Chronic alcohol intoxication and cortical ischemia: study of their comorbidity and the protective effects of minocycline. *Oxidative Medicine and Cellular Longevity*.

[23] Da Silva F. B., Cunha P. A., Ribera P. C. (2018). Heavy chronic ethanol exposure from adolescence to adulthood induces cerebellar neuronal loss and motor function damage in female rats. *Frontiers in Behavioral Neuroscience*.

[24] Oliveira A. C., Pereira M. C., Santana L. N. S. (2015). Chronic ethanol exposure during adolescence through early adulthood in female rats induces emotional and memory deficits associated with morphological and molecular alterations in hippocampus. *Journal of Psychopharmacology*.

[25] Belém-Filho I. J. A., Ribera P. C., Nascimento A. L. (2018). Low doses of methylmercury intoxication solely or associated to ethanol binge drinking induce psychiatric-like disorders in adolescent female rats. *Environmental Toxicology and Pharmacology*.

[26] Oliveira A. N., Pinheiro A. M., Belém-Filho I. J. A. (2018). Unravelling motor behaviour hallmarks in intoxicated adolescents: methylmercury subtoxic-dose exposure and binge ethanol intake paradigm in rats. *Environmental Science and Pollution Research*.

[27] Fernandes L. M. P., Cartágenes S. C., Barros M. A. (2018). Repeated cycles of binge-like ethanol exposure induce immediate and delayed neurobehavioral changes and hippocampal dysfunction in adolescent female rats. *Behavioural Brain Research*.

[28] Fernandes L. M. P., Lopes K. S., Santana L. N. S. (2018). Repeated Cycles of Binge-Like Ethanol Intake in Adolescent Female Rats Induce Motor Function Impairment and Oxidative Damage in Motor Cortex and Liver, but Not in Blood. *Oxidative Medicine and Cellular Longevity*.

[29] Pamplona-Santos D., Lamarão-Vieira K., Nascimento P. C. (2019). Aerobic physical exercise as a neuroprotector strategy for ethanol binge- drinking effects in the hippocampus and systemic redox status in rats. *Oxidative Medicine and Cellular Longevity*.

[30] Nascimento C. P., Luz D. A., da Silva C. C. S. (2020). Ganoderma lucidum ameliorates neurobehavioral changes and oxidative stress induced by ethanol binge drinking. *Oxidative Medicine and Cellular Longevity*.

[31] Maia C. D., Queiroz L. Y., de Oliveira I. G. (2021). Binge-like exposure during adolescence induces detrimental effects in alveolar bone that persist in adulthood. *Alcoholism, Clinical and Experimental Research*.

[32] Spear L. P. (2000). The adolescent brain and age-related behavioral manifestations. *Neuroscience and Biobehavioral Reviews*.

[33] Karl T., Pabst R., von Hörsten S. (2003). Behavioral phenotyping of mice in pharmacological and toxicological research. *Experimental and Toxicologic Pathology*.

[34] Tirelli E., Laviola G., Adriani W. (2003). Ontogenesis of behavioral sensitization and conditioned place preference induced by psychostimulants in laboratory rodents. *Neuroscience and Biobehavioral Reviews*.

[35] Semple B. D., Blomgren K., Gimlin K., Ferriero D. M., Noble-Haeusslein L. J. (2013). Brain development in rodents and humans: identifying benchmarks of maturation and vulnerability to injury across species. *Progress in Neurobiology*.

[36] Bahi A. (2013). Individual differences in elevated plus-maze exploration predicted higher ethanol consumption and preference in outbred mice. *Pharmacology, Biochemistry, and Behavior*.

[37] Kaster M. P., Machado N. J., Silva H. B. (2015). Caffeine acts through neuronal adenosine A2Areceptors to prevent mood and memory dysfunction triggered by chronic stress. *Proceedings of the National Academy of Sciences of the United States of America*.

[38] Sturman O., Germain P. L., Bohacek J. (2018). Exploratory rearing: a context- and stress-sensitive behavior recorded in the open-field test. *The International Journal on the Biology of Stress*.

[39] Pires V. A., Pamplona F. A., Pandolfo P., Fernandes D., Prediger R. D. S., Takahashi R. N. (2009). Adenosine receptor antagonists improve short-term object-recognition ability of spontaneously hypertensive rats: a rodent model of attention-deficit hyperactivity disorder. *Behavioural Pharmacology*.

[40] Handley S. L., Mithani S. (1984). Effects of alpha-adrenoceptor agonists and antagonists in a maze-exploration model of ?fear?-motivated behaviour. *Naunyn-Schmiedeberg’s Archives of Pharmacology*.

[41] Maia C. D., de Souza Lucena G. M., Corrêa P. B. (2009). Interference of ethanol and methylmercury in the developing central nervous system. *Neurotoxicology*.

[42] Castro N. C., Silva I. S., Cartágenes S. C. (2022). Morphine perinatal exposure induces long-lasting negative emotional states in adult offspring rodents. *Pharmaceutics*.

[43] Pellow S., Chopin P., File S. E., Briley M. (1985). Validation of open : closed arm entries in an elevated plus-maze as a measure of anxiety in the rat. *Journal of Neuroscience Methods*.

[44] Cryan J. F., Markou A., Lucki I. (2002). Assessing antidepressant activity in rodents: recent developments and future needs. *Trends in Pharmacological Sciences*.

[45] Alcaro A., Cabib S., Ventura R., Puglisi-Allegra S. (2002). Genotype- and experience-dependent susceptibility to depressive-like responses in the forced-swimming test. *Psychopharmacology*.

[46] Petit-Demouliere B., Chenu F., Bourin M. (2005). Forced swimming test in mice: a review of antidepressant activity. *Psychopharmacology*.

[47] Miller N. J., Rice-Evans C., Davies M. J. (1993). A new method for measuring antioxidant activity. *Biochemical Society Transactions*.

[48] Re R., Pellegrini N., Proteggente A., Pannala A., Yang M., Rice-Evans C. (1999). Antioxidant activity applying an improved ABTS radical cation decolorization assay. *Free Radical Biology and Medicine*.

[49] Vasconcelos S. M. L., Goulart M. O. F., Moura J. B., Manfredini V., Benfato M. . S., Kubota L. T. (2007). Espécies reativas de oxigênio e de nitrogênio, antioxidantes e marcadores de dano oxidativo em sangue humano: principais métodos analíticos para sua determinação. *Química Nova*.

[50] Ellman G. L. (1959). Tissue sulfhydryl groups. *Archives of Biochemistry and Biophysics*.

[51] Granger D. L., Anstey N. M., Miller W. C., Weinberg J. B. (1999). [6] Measuring nitric oxide production in human clinical studies. *Methods in Enzymology*.

[52] Percario S. (1994). Dosagem das LDLs modificadas através da peroxidação lipídica: correlação com o risco aterogênico. *Anais Medicos dos Hospitais e da Faculdade de Ciências Médicas da Santa Casa de São Paulo*.

[53] da Silveira C. C. S., Fernandes L. M. P., Silva M. L. (2016). Neurobehavioral and antioxidant effects of ethanolic extract of yellow propolis. *Oxidative Medicine and Cellular Longevity*.

[54] Kohn H. I., Liversedge M. (1994). On a new aerobic metabolite whose production by brain is inhibited by apomorphine, emetine, ergotamine, epinephrine, and menadione. *Journal of Pharmacology and Experimental Therapeutics*.

[55] Ferreira W. A. S., Amorim C. K. N., Burbano R. R. (2021). Genomic and transcriptomic characterization of the human glioblastoma cell line AHOL1. *Brazilian Journal of Medical and Biological Research*.

[56] Bertotto M. E., Bustos S. G., Molina V. A., Martijena I. D. (2006). Influence of ethanol withdrawal on fear memory: effect of d-cycloserine. *Neuroscience*.

[57] De Witte P., Pinto E., Ansseau M., Verbanck P. (2003). Alcohol and withdrawal: from animal research to clinical issues. *Neuroscience and Biobehavioral Reviews*.

[58] Montesinos J., Pascual M., Rodríguez-Arias M., Miñarro J., Guerri C. (2016). Involvement of TLR4 in the long-term epigenetic changes, rewarding and anxiety effects induced by intermittent ethanol treatment in adolescence. *Brain, Behavior, and Immunity*.

[59] Lal H., Prather P. L., Rezazadeh S. M. (1991). Anxiogenic behavior in rats during acute and protracted ethanol withdrawal: reversal by buspirone. *Alcohol*.

[60] Gatch M. B., Wallis C. J., Lal H. (1999). Effects of NMDA antagonists on ethanol-withdrawal induced "anxiety" in the elevated plus maze. *Alcohol*.

[61] Gonzaga N. A., Mecawi A. S., Antunes-Rodrigues J., de Martinis B. S., Padovan C. M., Tirapelli C. R. (2015). Ethanol withdrawal increases oxidative stress and reduces nitric oxide bioavailability in the vasculature of rats. *Alcohol*.

[62] Pandey S. C., Sakharkar A. J., Tang L., Zhang H. (2015). Potential role of adolescent alcohol exposure-induced amygdaloid histone modifications in anxiety and alcohol intake during adulthood. *Neurobiology of Disease*.

[63] Valdez G. R., Roberts A. J., Chan K. (2002). Increased ethanol self-administration and anxiety-like behavior during acute ethanol withdrawal and protracted abstinence: regulation by corticotropin- releasing factor. *Alcoholism, Clinical and Experimental Research*.

[64] Hershon H. I. (1977). Alcohol withdrawal symptoms and drinking behavior. *Journal of Studies on Alcohol*.

[65] Badanich K. A., Maldonado A. M., Kirstein C. L. (2007). Chronic ethanol exposure during adolescence increases basal dopamine in the nucleus accumbens septi during adulthood. *Alcoholism, Clinical and Experimental Research*.

[66] Pascual M., Boix J., Felipo V., Guerri C. (2009). Repeated alcohol administration during adolescence causes changes in the mesolimbic dopaminergic and glutamatergic systems and promotes alcohol intake in the adult rat. *Journal of Neurochemistry*.

[67] Rossetti Z. L., Melis F., Carboni S., Diana M., Gessa G. L. (1992). Alcohol withdrawal in rats is associated with a marked fall in extraneuronal dopamine. *Alcoholism, Clinical and Experimental Research*.

[68] Sircar R. (2017). Ethanol alters N-methyl-D-aspartate receptor regulation in the hippocampus of adolescent rats. *Neuroreport*.

[69] Marco E. M., Peñasco S., Hernández M. D. (2017). Long-term effects of intermittent adolescent alcohol exposure in male and female rats. *Frontiers in Behavioral Neuroscience*.

[70] Vetreno R. P., Crews F. T. (2015). Binge ethanol exposure during adolescence leads to a persistent loss of neurogenesis in the dorsal and ventral hippocampus that is associated with impaired adult cognitive functioning. *Frontiers in Neuroscience*.

[71] Yang J. Y., Xue X., Tian H. (2014). Role of microglia in ethanol-induced neurodegenerative disease: pathological and behavioral dysfunction at different developmental stages. *Pharmacology & Therapeutics*.

[72] Salim S. (2017). Oxidative stress and the central nervous system. *The Journal of Pharmacology and Experimental Therapeutics*.

[73] Forman H. J., Zhang H., Rinna A. (2009). Glutathione: overview of its protective roles, measurement, and biosynthesis. *Molecular Aspects of Medicine*.

[74] Wu D., Cederbaum A. I. (2009). Oxidative stress and alcoholic liver disease. *Seminars in Liver Disease*.

[75] Meister A. (1982). Metabolism and function of glutathione: an overview. *Biochemical Society Transactions*.

[76] Muñiz-Hernández S. (2012). Alcoholism: common and oxidative damage biomarkers. *Journal of Clinical Toxicology*.

[77] Montoliu C., Vallés S., Renau-Piqueras J., Guerri C. (1994). Ethanol-induced oxygen radical formation and lipid peroxidation in rat brain: effect of chronic alcohol consumption. *Journal of Neurochemistry*.

